# Supplementary material for: HIV and antiretroviral treatment knowledge gaps and psychosocial burden among persons living with HIV in Lima, Peru
Source: PLoS One. 2021 Aug 19;16(8):e0256289. doi: 10.1371/journal.pone.0256289 (PMC8376082; doi:10.1371/journal.pone.0256289)
Supplement: S1 Table — (DOCX) [file pone.0256289.s001.docx]

**S1: Score used to quantify Knowledge on HIV and on ARV**

|  | **Correct**  **Answer** | **Score (points)** |
| --- | --- | --- |
| **Knowledge on HIV** |  |  |
| I must use a condom in any sexual relation with a person without HIV* | True | 0.25 |
| Is HIV an illness that can be cured or controlled? | Controlled | 0.25 |
| If I use condoms correctly I will have safe sex | True | 0.25 |
| I have to use a condom if I have sex with a PLWH* | True | 0.25 |
| I am at risk of another infection if I have sex with a PLWH without using a condom | True | 0.25 |
| A PLWH can live the same number of years as a person not infected? | Yes | 0.25 |
| HIV infection places me at higher risk of |  |  |
| Diarrhea | True | 0.25 |
| Sexually transmitted infections | True | 0.25 |
| Dental problems | True | 0.25 |
| Cancer | True | 0.25 |
| The use of microbicides during sex avoids HIV transmission | False | 0.25 |
| **Knowledge on ARV** |  |  |
| If I am feeling ok, I can discontinue ARV | False | 1 |
| It is important to discontinue ARV for a few days to rest the body | False | 1 |
| While I am on ARV, I do not transmit HIV | False | 1 |
| If I forget to take my ARV, I can make up for it by taking a double dose the following day | False | 1 |
| The following substances interfere with ARV |  |  |
| Cocaine | True | 0.25 |
| Large amounts of alcohol | True | 0.25 |
| Marijuana | True | 0.25 |

PLWH= Persons living with HIV; ARV= Antiretrovirals
