# Supplementary material for: HIV and antiretroviral treatment knowledge gaps and psychosocial burden among persons living with HIV in Lima, Peru
Source: PLoS One. 2021 Aug 19;16(8):e0256289. doi: 10.1371/journal.pone.0256289 (PMC8376082; doi:10.1371/journal.pone.0256289)
Supplement: Attachment — Submitted filename: Response to Reviewers.doc [file pone.0256289.s003.doc]

**RESPONSE TO REVIEWER´S COMMENTS**

**Manuscript PONE-D-21-01332**

**HIV and antiretroviral treatment knowledge gaps and psychosocial burden among persons living with HIV in Lima, Peru**

**Reviewer's Responses to Questions**

**1. Is the manuscript technically sound, and do the data support the conclusions?**

**Reviewer #1: Yes Reviewer #2: Yes**

Thank you for the positive review

**2. Has the statistical analysis been performed appropriately and rigorously?**

**Reviewer #1: Yes Reviewer #2: Yes**

Thank you for the positive review

**3. Have the authors made all data underlying the findings in their manuscript fully available?**

**Reviewer #1: No	Reviewer #2: No**

The full database is now fully available. The data does not have any identifiable information.

**4. Is the manuscript presented in an intelligible fashion and written in standard English?**

**Reviewer #1: Yes Reviewer #2: No**

Thank you noticing this. A native English speaker has reviewed our paper and edited the grammar of the text.

**Review Comments to the Author**

**Reviewer #1: A well-written paper that provides information about the levels of knowledge of HIV and ARV treatment as well as the prevalence of psycho-social factors among consenting PLWH waiting for viral load measurement at the waiting room of a tertiary healthcare institution in Lima, Peru.**

Thank you for the positive review.

**To further improve the paper quality the following are suggested,**

1. **The title should be revised to include the fact that the participants were consenting to PLWH at the waiting room of the hospital for viral load measurement. This is necessary as there is no sample size estimation and the representativeness, as well as the generalisability of the findings, is questionable.**

Thank you for the observation. We agree with the recommendation and modified the title so it reads: *HIV and antiretroviral treatment knowledge gaps and psychosocial burden among persons living with HIV linked to care in Lima, Peru.* We now describe the sample size estimation on page 5 line 104-107 (version without tracked changes).

2. **The rationale for this work needs to be further clarified. The authors mentioned that the level of HIV related knowledge among PLWH has not been quantified, but cited studies that indicated that PLWH understood all information given by their doctors. Besides, qualitative studies on HIV transmission reveals that misconception on HIV transmission exists. The authors should indicate the gap in knowledge that this study was out to bridge.**

We have reviewed the text (page 3 line 72-79) to clarify the gap in knowledge that justifies the purpose of this study. The text now reads:

*In Lima, Peru, although the level of HIV and ARV knowledge among PLWH has not been fully quantified, some studies have shed light on health information among PLWH. In a study in Lima, 51.1% of PLWH said that they were satisfied with all information given by the doctors [16] and a qualitative study in Piura, Peru concluded that PLWH on ARV have important misconceptions on HIV transmission and treatment. [17] Yet, the specific HIV and ARV knowledge gaps among PLWH have not been described, which is necessary to design educational interventions.*

3. **The authors recognised the importance of adherence to ARV as a key to sustained viral suppression in HIV control in their introduction yet did not measure this important variable by any means from the participants in the study. It would have been good to know the role of adherence as a potential confounder or middle variable in the association between viral suppression and knowledge or psychosocial factors.**

Thank you for noticing this. In fact, adherence to ARV is key to sustained viral suppression in HIV control and is important to measure, but this data is not available in the studys` hospital. We have included the importance of systematically measuring adherence in this hospital (and in all health care centers in Peru).

4. **The aspect of the paper which sought to analyse and proved information about the "impact of HIV and ARV knowledge on viral suppression" is not methodologically adequate given the unmeasured adherence level among participants, different samples sizes used for knowledge assessment and viral suppression, and the fact that it's not included as an objective of this study. I, therefore, suggest that the portion be expunged from the paper.**

We agree that without controlling for adherence, we cannot measure the effect of knowledge on viral suppression. We have taken out that analysis from the manuscript.

Thank you for bringing this up.

**Reviewer #2: The content of the manuscript is scientifically relevant and the findings are in line with similar papers coming out of this region.**

Thank you for the positive review.

**There are certain issues which I would like to point out…**

1. **The language has to be considerably improved throughout the manuscript. I would suggest seeking help from an English language editor.**

We have taken your suggestion into account and requested a native English speaker to review our paper and made grammar changes in the text.

2. **Sample size calculation and sampling strategy are not mentioned in the manuscript. Were the sample universal and only those fulfilling the inclusion criteria were included?**

Thank you for your observation. We have included sample size estimation on page 4 line 93-96 so it reads:

*Expecting 15% of participants to have low knowledge [5] with a confidence level of 95% and a precision of 5% for a population size of 1580 (PLWH enrolled in the HIV program) and expecting 15% of non-response, we estimated a sample size of 175 participants.*

3. **The scoring assigned for the knowledge section of the questionnaire is not clearly explained and is quite confusing.**

Thank you. We have now clarified the explanation of our score and we clarified it on page 6. Also, we have added a complimentary table (S1 Table) with each question and its correct answer and corresponding score on page 24.

4. **What is the difference between does not know and do not answer ---is the difference literal?**

Each question of our questionnaire had the option *I don`t know* and the self-administered paper survey made it possible for the participant to leave some questions unanswered.

5. **I would suggest retaining the correct responses and arrange them in descending order.**

As suggested, we have restructured table 1 on page 9.

6. **Some of the statements under Knowledge regarding ARV are assessing perception, rather than knowledge.**

Thank you for bringing this up. Despite the fact that some statements could be interpreted as perceptions. In reality, they are measuring knowledge. For example, the statement “While I am on ARV, I do not transmit HIV” measures the knowledge of participants regarding HIV transmissibility while being on ARVs. Also, previous studies (Van Servellen, 2003; Kalichman, 2008) have used similar questions to ours to describe ARV knowledge.

7. **Class interval chosen for age groups are not uniform? what is the basis for such interval?**

We use quartiles to made our age groups. We have included this information in methods on page 6 line 144-146.

8. **For tables determining the factors for not achieving viral suppression, the information is non-coherent with loads of variables. The interpretation becomes too difficult.**

The table that presented the bivariate and multivariate analysis of factors for not achieving viral suppression is no longer part of the manuscript.
